# TP53 mutation-mediated genomic instability induces the evolution of chemoresistance and recurrence in epithelial ovarian cancer

**DOI:** 10.1186/s13000-017-0605-8

**Published:** 2017-02-02

**Authors:** Meiying Zhang, Guanglei Zhuang, Xiangjun Sun, Yanying Shen, Wenjing Wang, Qing Li, Wen Di

**Affiliations:** 1 Department of Obstetrics and Gynecology, Ren Ji Hospital, School of Medicine, Shanghai Jiao Tong University, 160 Pujian Road, Shanghai, 200127 China; 2 Shanghai Key Laboratory of Gynecologic Oncology, Ren Ji Hospital, School of Medicine, Shanghai Jiao Tong University, 160 Pujian Road, Shanghai, 200127 China; 3 State Key Laboratory of Oncogenes and Related Genes, Renji-Med X Clinical Stem Cell Research Center, Ren Ji Hospital, School of Medicine, Shanghai Jiao Tong University, 160 Pujian Road, Shanghai, 200127 China; 4 Department of Pathology, Ren Ji Hospital, School of Medicine, Shanghai Jiao Tong University, 160 Pujian Road, Shanghai, 200127 China

**Keywords:** Epithelial ovarian cancer, *TP53* mutation, Genomic instability, *MDR1* copy number, Recurrence

## Abstract

**Background:**

Genomic instability caused by mutation of the checkpoint molecule *TP53* may endow cancer cells with the ability to undergo genomic evolution to survive stress and treatment. We attempted to gain insight into the potential contribution of ovarian cancer genomic instability resulted from *TP53* mutation to the aberrant expression of multidrug resistance gene *MDR1*.

**Methods:**

*TP53* mutation status was assessed by performing nucleotide sequencing and immunohistochemistry. Ovarian cancer cell DNA ploidy was determined using Feulgen-stained smears or flow cytometry. DNA copy number was analyzed by performing fluorescence in situ hybridization (FISH).

**Results:**

In addition to performing nucleotide sequencing for 5 cases of ovarian cancer, *TP53* mutations were analyzed via immunohistochemical staining for P53. Both intensive P53 immunohistochemical staining and complete absence of signal were associated with the occurrence of *TP53* mutations. HE staining and the quantification of DNA content indicated a significantly higher proportion of polyploidy and aneuploidy cells in the *TP53* mutant group than in the wild-type group (*p* < 0.05). Moreover, in 161 epithelial ovarian cancer patients, multivariate logistic analysis identified late FIGO (International Federation of Gynecology and Obstetrics) stage, serous histotype, G3 grade and *TP53* mutation as independent risk factors for ovarian cancer recurrence. In relapse patients, the proportion of chemoresistant cases in the *TP53* wild-type group was significantly lower than in the mutant group (63.6% vs. 91.8%, *p* < 0.05). FISH results revealed a higher percentage of cells with >6 *MDR1* copies and chromosome 7 amplication in the *TP53* mutant group than in the wild-type group [11.7 ± 2.3% vs. 3.0 ± 0.7% and 2.1 ± 0.7% vs. 0.3 ± 0.05%, (*p* < 0.05), respectively]. And we observed a specific increase of *MDR1* and chromosome 7 copy numbers in the *TP53* mutant group upon disease regression (*p* < 0.01).

**Conclusions:**

*TP53* mutation-associated genomic instability may promote chromosome 7 accumulation and *MDR1* amplification during ovarian cancer chemoresistance and recurrence. Our findings lay the foundation for the development of promising chemotherapeutic approaches to treat aggressive and recurrent ovarian cancer.

## Background

Genomic evolution is one of the basic features of human cancers and endows malignant cells with the ability to survive and tolerate rigorous microenvironments, such as anoxia, ischemia and starvation, as well as resist attacks from immune cells [1–4]. Therefore, during chemotherapy, cancer cells undergo genomic changes to adapt to chemotherapeutics, a phenomenon referred to as single- or multi-drug chemoresistance [5, 6]. According to Darwinian evolutionary theory, evolutionary process can be attributed to the presence of genetic mutations between parental and offspring generations [7]. Thus, somatic mutations, and the consequent genomic instability may be an important driving force for the development of chemoresistance in malignant tumors. Genomic instability is defined as an increased rate of DNA alterations [8]. *TP53* is a checkpoint molecule that maintains genomic stability, prevents cell mitosis and induces apoptosis following abnormal chromosome segregation or chemical damage to DNA sequences [9, 10]. The absence or mutation of *TP53* promotes two types of genomic instability, chromosomal and amplification instability, resulting in daughter cells that exhibit large amount of aneuploidy or abnormal chromosomes [11, 12]. Moreover, other related genes, such as pyruvate kinase isoform M2 (*PKM2*), breast cancer 1 (*BRCA1*) and homolog of the Schizosaccharomyces pombe cell cycle checkpoint gene (*RAD17*), are affected by genomic instability caused by *TP53* gene mutation, resulting in an improved microenvironment that facilitates the ability of a cell to endure [13, 14]. Thus far, *TP53* gene mutations have been verified in more than 50% ovarian cancer patients, primarily in patients with high-grade serous carcinoma whose prognosis is worse [15, 16]. In a recent rigorous reassessement, 100% of high-grade serous ovarian cancers exhibited *TP53* mutations [17].

The evolutionary behavior of ovarian cancer includes short-term recurrence after anti-cancer treatments, which is the primary reason for poor prognosis. This recurrence is not only closely linked to a late FIGO stage and a high pathological grade, but is also fueled by chemoresistance [18, 19]. *TP53* mutations impart oncogenic proprieties, as exemplified by genomic instability, the deregulation of cell cycle progression, and multiple chemotherapeutic resistance [13, 20]. In addition, overexpression of the multi-drug resistance gene MDR1 confers multi-drug resistance in almost all the solid tumors via the PI3K/Akt/Nrf2, Wnt/β-catenin, and HIF-1α/MDR1 pathways [21–24]. Along this line, it has been revealed that MDR1 and epithelial-mesenchymal transition (EMT) were present in the P53-null breast cancer cell line MCF-7, which was resistant to doxorubicin [25]. However, whether aberrant expression of the *MDR1* gene is associated with cancer genomic instability caused by *TP53* gene mutation remains unknown and requires in- depth exploration.

In the current study, we analyzed the relationship between *TP53* gene mutation and the evolution of chemoresistance in detail in ovarian cancer patients. We found that *TP53* mutation promoted chromosome aneuploidy and induced *MDR1* amplification instability. What’s more, these patients were prone to relapse after chemotherapy. Thus, our data advocated to formulate clinically individualized treatments for ovarian cancer patients.

## Methods

### Study population

A total of 161 epithelial ovarian cancer patients were recruited from the Department of Obstetrics and Gynecology, Ren Ji Hospital, Shanghai, China, between June 2003 and December 2009. Patients met the following criteria: no neoadjuvant chemotherapy, treatment with cytoreductive surgery, a diagnosis of epithelial ovarian cancer confirmed by pathology and a standardized platinum-based chemotherapy after surgery. This research was approved by the Ethics Committee (No. RJ2015-087 k) of Ren Ji Hospital, Shanghai Jiao Tong University, School of Medicine, and informed consents were obtained from all patients or their direct relatives. The following information was recorded: clinicopathological characteristics, chemoresistance to platinum and patient prognosis. Patients’ survival time was defined by the time from diagnosis until the date of death, or the last day of follow-up for surviving patients.

### *TP53* mutation analysis

Paraffin-embedded cancer specimens were dewaxed, rehydrated and stained with hematoxylin and eosin (HE) to identify the cancerous regions. An LMD6500 Laser Microdissection System (Leica, Wetzlar, Germany) was used to capture cancer cells, and DNA was extracted using a QIAamp DNA FFPE Tissue Kit (Qiagen, Valencia, CA, USA). DNA samples were then analyzed using a Qubit dsDNA HS Assay Kits (Life Technologies, Waltham, MA, USA). Targeted exon sequencing was performed with a MiSeq Reagent Kit v2 (Illumina, San Diego, CA, USA), which enables the detection of low levels of mutation from smaller amounts of DNA. Subsequently, we prepared amplified DNA libraries with a Gene Read DNA Libraries I Core Kit (Qiagen, Valencia, CA, USA), and all libraries were diluted to the designed range for cluster generation via the Illumina platform. Deep-sequencing was then performed using MiSeq (Illumina, San Diego CA, USA).

### Immunohistochemistry

Paraffin-embedded tumor tissue sections (4 μm) were collected on slides, and antigen retrieval was conducted by microwaving the samples at >90°C after they were dewaxed and rehydrated. Slides were then blocked with 5% bovine serum albumin (BSA) for 1 h to reduce non-specific binding. The specimens were incubated with the following primary antibodies for 2 h at 1:100 dilution: monoclonal mouse anti-human P53 (Clone DO-7, SANTA CRUZ, Dallas, Texas, USA) or monoclonal mouse anti-human MDR1 (SANTA CRUZ, Dallas, Texas, USA). Specimens were then incubated with a secondary antibody horseradish peroxidase (HRP)-conjugated goat anti-mouse lgG polyclonal antibody (Zhongshan, Beijing, China, dilution of 1:100) for 1 h. 3,3’-diaminobenzidine tetrahydrochloride (DAB; Zhongshan, Beijing, China) was used as a chromogen, and hematoxylin was used to counterstain the slides. Semiquantitative staining evaluation which included the fraction and intensity of stained cells, was performed by two pathologists in a blinded fashion.

### Quantitative analysis of cellular DNA

DNA smear image cytometry was performed on 20 patients with epithelial ovarian cancer. Deep-frozen cancer tissues were first cut into 8 μm-thick frozen sections. Laser capture microdissection was performed to dissect the cancer islets, which were digested with 0.25% trypsin (Biyuntian, Beijing, China) and 0.2% collagenase IV (Sigma-Aldrich, St Louis, MO, USA). Samples were filtered through a 70 μm screen mesh, fixed for 1 h in Bohm-Sprenger liquid (Mike Audi Corporation, Xiamen, China) and smeared to prepare single-cell-layer slides. The slides were then stainedwith Feulgen DNA dye (Mike Audi Corporation, Xiamen, China) for 75 min. DNA images were captured with a microscope, and data were analyzed withMotiCytometer and MotiClassify software (Mike Audi Corporation, Xiamen, China).

### Cell cycle analysis

We collected additional fresh cancer tissue samples from 50 patients, microdissected the cancerous regions and digested the samples as described aboveto prepare ovarian cancer cell suspensions. Cells were then fixed with 70% ethanol overnight, and stained with propidium iodide (PI, 10 μg/ml) containing RNase (1 mg/ml) at 4°C for 30 min. Flow cytometry analysis of cellular DNA content was performed using a FC500 MPL flow cytometer (Beckman Coulter, Brea, CA, USA) with a total of 10,000 events per sample.

### Fluorescence in situ hybridization

After paraffin-embedded tumor slides were dewaxed, fixed and rehydrated, samples were denatured via microwave exposure, and digested with pepsin (Sigma-Aldrich, St Louis, MO, USA). Then, slides were labeled with MDR1/CEN7 (centromere of chromosome 7) dual-fluorescent probes (Zytolight, Bremerhaven, Germany), mounted with rubber cement (Best-Test, Galesburg, IL, USA), and allowed to hybridize at 37°C overnight. The next day, the slides were stained with DAPI and analyzed with a Leica DM2500 fluorescence microscope.

### Statistic analysis

Differences in survival based on clinicopathological characteristics were assessed using a log-rank test. Correlations between the presence of *TP53* mutation and clinicopathological factors were analyzed using Fisher’s exact test, while nucleus diameter, DNA index and copy number were analyzed using Student’s *t*-test. The relationships among ovarian cancer clinicopathological characteristics, *TP53* mutation and recurrence were analyzed by performing multivariate logistic regression analysis. All analyses were performed with SPSS 19.0 software, and *p* < 0.05 was considered statistically significant.

## Results

### The relationship between the presence of *TP53* mutation and patient clinicopathological characteristics

**Fig. 1:**
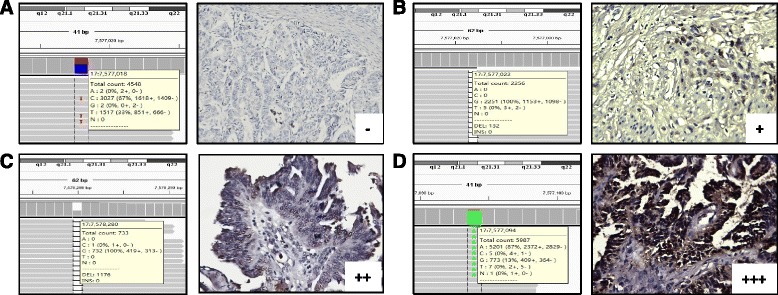
Immunohistochemistry results for 4 ovarian cancer samples harboring *TP53* mutations. **a**: the patient with a *TP53* nonsense mutation exhibited negative P53 staining; **b**: the patient with a low abundance (5.7%) of *TP53* frameshift mutation exhibited faint P53 staining; **c**: the patient with a *TP53* frameshift mutation exhibited moderate P53 staining; **d**: the patient with a *TP53* missense mutation exhibited strong P53 staining. (×400)

This study included 161 epithelial ovarian cancer patients recruited from the Department of Obstetrics and Gynecology at Shanghai Ren Ji Hospital. To confirm P53 immunostaining as a surrogate marker for the presence of *TP53* mutation [26, 27], *TP53* nucleotide sequencing and P53 immunostaining results were compared on samples obtained from 5 serous ovarian cancer patients. Next-generation sequencing analysis identified non-synonymous *TP53* mutations in 4 high-grade patients, while 1 low-grade patient exhibited wild-type *TP53* (Table 1). Of the 4 patients with mutant *TP53,* 1 patient contained a nonsense mutation leading to the complete disruption of P53 expression, evidenced by negative staining for P53, which was referred to as a class I mutation. 2 patients exhibited a missense mutation or a frameshift mutation, resulting in partially damaged P53 function and a strong positive signal of P53 staining (moderate-to-strong staining cells in greater than 60% fraction), referred to as a class II mutation. Additionally, 1 patient exhibited a class III mutation comprising a frameshift mutation with <10% *TP53* mutation abundance. Immunohistochemistry showed positive staining for P53 (10%-50% moderate staining cells) (Fig. 1). The patient with wild-type *TP53* displayed weak positive staining for P53 (<10% positive cells). As the patient with a class III mutation contained only a small amount of *TP53* mutant tissue within the entire tumor, and nearly unimpaired P53 protein function, this sample was approximately classified into the *TP53* wild-type group, whereas class I and class II mutations were considered as the *TP53* mutant group. Based on the results of the above analysis, P53 IHC may be used as a surrogate marker of *TP53* mutation, and immunohistochemistry was performed for all 161 patients to identify the presence of *TP53* mutation in our subsequent studies. A total of 123 patients harbored mutant *TP53*, while 38 patients exhibited wild-type *TP53*. The clinical characteristics of these patients are listed in Table 2. Parity, peritoneal metastasis, FIGO stage, tumor grade, CA125 level, the presence of residual tumor, and chemoresistance to platinum were related to the prognosis of ovarian cancer patients. Moreover, *TP53* mutant patients tended to have low parity, increased peritoneal metastasis, late FIGO stage and high tumor grade.

**Table 1:** DNA Sequencing and IHC Results

Sample	*TP53* Mutation by Sequencing	Class of Mutation	IHC Results	Proportion of Tumor cells (%)
1 2 3 4 5	R306 (abundance: 61.5%) P190 (abundance: 5.7%) Negative Exon 8 C > T (abundance: 33.8%) R282W (abundance: 88.4%)	II III Wild-type I II	++ + ± - +++	80 90 75 85 80

*IHC* immunohistochemistry

P53 nuclei staining: -, complete absence of staining; ±, <10% of tumor cells with faint staining intensity; +, 10%-50% of tumor cells with faint staining intensity; ++, ≥50% of tumor cells with moderate staining intensity; +++, ≥50% of tumor cells with strong staining intensity

**Table 2:** Clinicopathological Characteristics and *TP53* Mutational Analysis

Characteristics	Number of total patients	(%)	P^c^	Number of *TP53* Mutations	(%)	P^d^
Parity			0.019*			0.042*
0-1	77	47.8		59	48.0	
2-3	66	41.0		57	46.3	
>3	15	9.3		7	5.7	
Peritoneal metastasis			0.004*			0.027*
yes	89	55.3		79	64.2	
no FIGO stage	71	44.1	0.025*	44	35.8	0.024*
I	55	34.2		33	26.8	
II	18	11.2		17	13.8	
III	80	49.7		66	53.7	
IV	7	4.3		7	5.7	
Histotype			0.954			0.114
serous	91	56.5		70	56.9	
mucinous	22	13.7		16	13.0	
endometrioid	23	14.3		13	10.6	
clear cell	16	9.9		15	12.2	
undifferentiated	9	5.6		9	7.3	
WHO grade			0.415			0.068
G1	33	20.5		22	17.9	
G2	57	35.4		39	31.7	
G3	69	42.9		62	50.4	
Tumor grade^a^			0.003*			0.002*
low-grade	57	35.4		33	26.8	
high-grade	101	62.7		90	73.2	
CA125 (Uml^–1^) ^b^			0.001*			0.508
≤ 206.52	86	53.4		51	41.5	
> 206.52	46	28.6		39	31.7	
Residual tumor (cm)			<0.0001*			0.102
≤ 0.5	119	73.9		86	69.9	
> 0.5	40	24.8		37	30.1	
Platinum resistance			<0.0001*			0.161
yes	36	22.4		33	26.8	
no	123	76.4		90	73.2	

a: In accordance with morphological and molecular genetic analysis, EOC was divided into two categories [28]

b: Based on patient survival, the cutoff value of CA125 calculated with a receiver operating characteristic (ROC) was 206.52 (Uml–1)

c: *p* value for clinicopathological characteristics

d: *p* value for *TP53* mutation

*p < 0.05

### The analysis of *TP53* mutations and patient prognosis

**Fig. 2:**
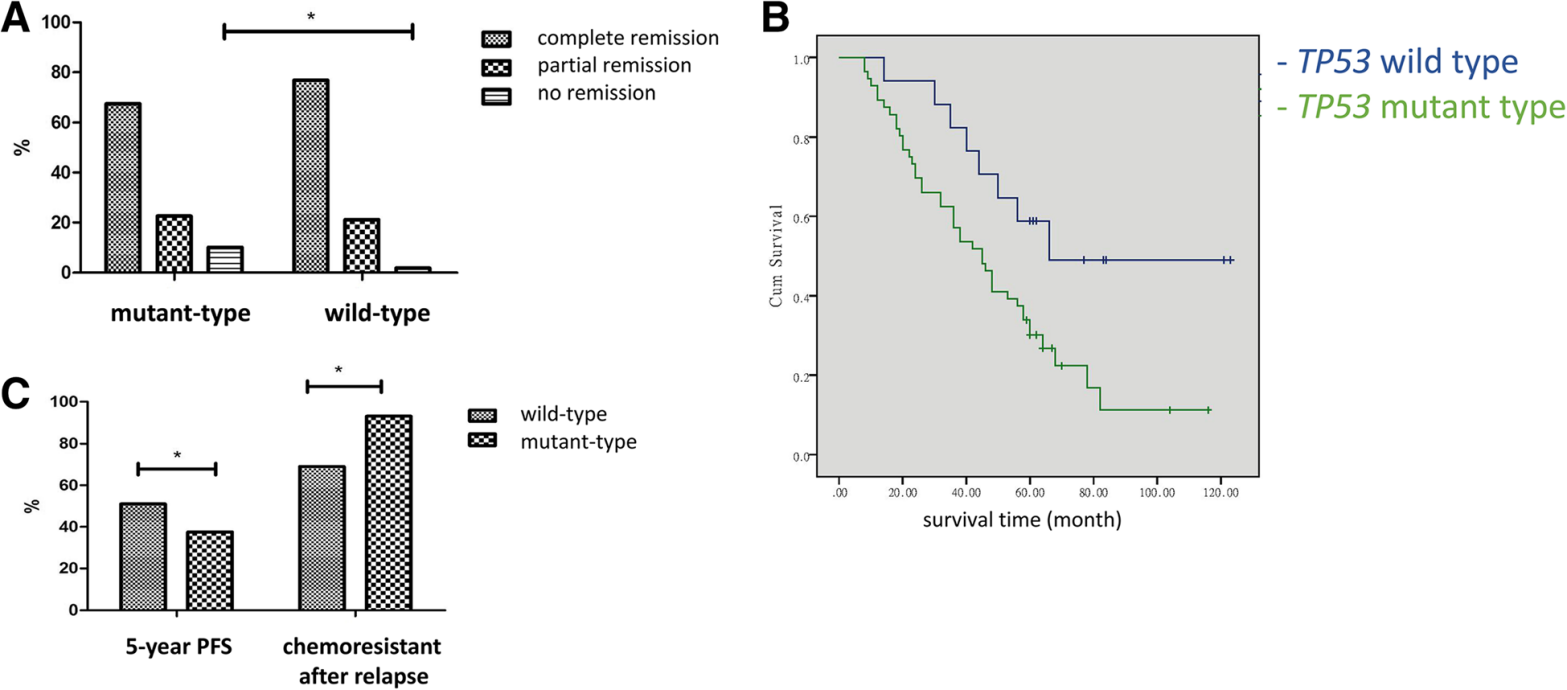
The relationship between the presence of *TP53* mutation and ovarian cancer patient prognosis. **a**: proportional analysis of complete, partial and no remission after the first round of chemotherapy between the *TP53* mutant and wild-type groups; **b**: Kaplan-Meier analysis of the *TP53* mutant and wild-type groups (*p* < 0.05); **c**: the ratio of 5-year progression-free survival and subsequent chemoresistance after relapse between the *TP53* mutant and wild-type groups. *p < 0.05

After chemotherapy, in the *TP53* mutant group, the percentage of patients demonstrating complete, partial and no remission was 67.5% (83/123), 22.8% (28/123) and 9.7% (12/123), respectively, while in the wild-type group, the percentage of complete, partial and no remission was 76.3% (29/38), 21.1% (8/38) and 2.6% (1/38), respectively. There was a significant difference (*p* < 0.01) in the number of no remission patients between the two groups (Fig. 2a). Kaplan-Meier analysis revealed longer overall survival (OS) of patients in the *TP53* wild-type group (82.59 ± 10.50 months) than those in the mutant group (49.41 ± 4.72 months) (*p* < 0.05) (Fig. 2b). The 5-year survival rates were not statistically significantly different between the wild-type group (58.2%) and the mutant group (46.7%), and multivariate analysis revealed late stage, the presence of serous cancer and G3 grade to be independent predictive factors for 5-year survival. In patients with complete remission, the rate of 5-year progression-free survival (PFS) in the wild-type group (51.7%, 15/29) was higher than in the mutant group (37.3%, 31/83) (*p* < 0.05) (Fig. 2C). In addition, in relapse patients, stages IIb ~ IIIc accounted for 83.3% (60/72) of patients, serous cancer was present in 55.6% (40/72) of patients,G3 grade was detected in 51.4% (37/72) of patients and *TP53* mutations (class I/II) were evident in 84.7% (61/72) of patients. Multivariate logistic analysis revealed late FIGO stage, serous cancer, G3 grade and *TP53* mutation to be independent factors of ovarian cancer recurrence. The proportion of cases resistant to TP (paclitaxel and cisplatin) chemotherapy (including partial and no remission) after relapse in the wild-type group was 63.6% (7/11), compared to 91.8% (56/61) in the mutant group (*p* < 0.05) (Fig. 2c).

### The relationship between *TP53* gene mutation and DNA abnormalities in cancer cell nuclei

**Fig. 3:**
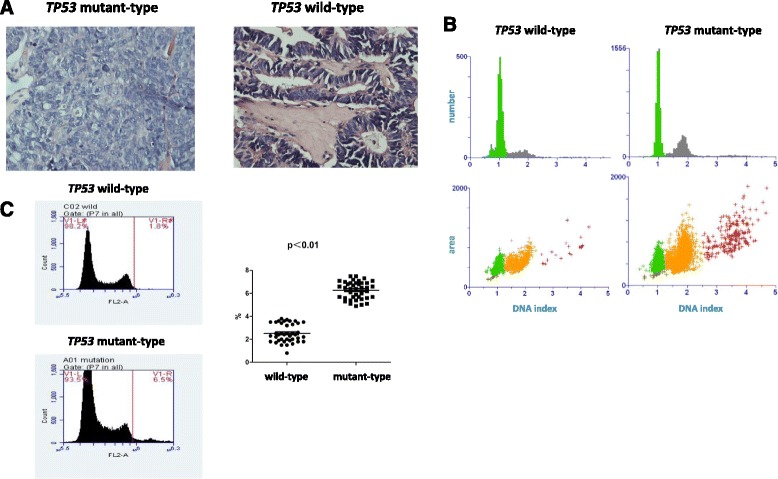
Analysis of *TP53* mutations and DNA abnormalities. **a**: HE staining of the *TP53* wild-type and mutant ovarian cancer cells to investigate nucleus morphology; **b**: quantitative analysis of the ovarian cancer cell DNA index in the *TP53* wild-type and mutant groups; **c**: cell cycle analysis of polyploid cell ratios in two groups by flow cytometry

HE staining revealed the relative uniformity of cancer cell nucleus size in the *TP53* wild-type group, and the ratio of the largest and smallest nuleus diameters was 3.4 ± 0.3, which was remarkably smaller than the diameter ratio of 5.4 ± 0.8 for the mutant group (*p* < 0.05) (Fig. 3a). Quantitative detection of the DNA index with DNA-specific dye was performed in ovarian cancer cell smears from 20 epithelial ovarian cancer patients. The incidence of polyploidy (>2-fold) and aneuploidy (1.1-1.9-fold) in the mutant group was 78.2 ± 7.0%, which was significantly higher than that in the wild-type group (56.0 ± 3.8%, p < 0.05) (Fig. 3b). In addition, PI was used to stain ovarian cancer cell nuclei in samples from 50 patients, and the incidence of polyploidy and aneuploidy in the *TP53* mutant group was much higher than that in the wild-type group (*p* < 0.01) (Fig. 3c).

### The correlation between *TP53* mutation and *MDR1* copy number aberration

**Fig. 4:**
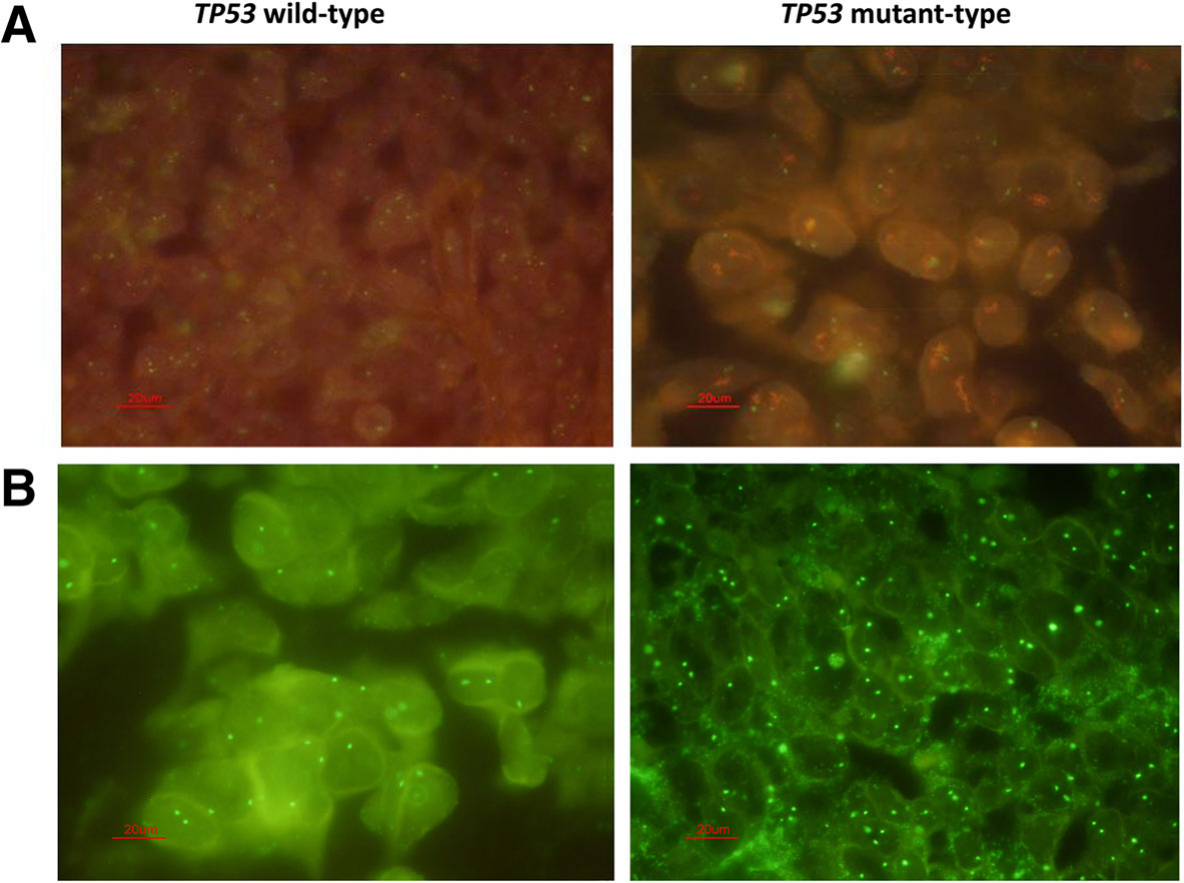
*MDR1* and chromosome 7 copy numbers were detected via FISH in two group samples. **a**: MDR1-FISH revealed *MDR1* copy numbers in the two groups, red indicates *MDR1*, and chromosome 7 centromeres are stained in green; **b**: the green-staining for chromosome 7 centromeres in the two groups

MDR1-FISH revealed the proportions of 5-6 copy and >6 copy cells to be 33.0 ± 5.6% and 3.0 ± 0.7%, respectively, in the *TP53* wild-type group, which were significantly lower than those in the mutant group (37.8 ± 7.9% and 11.7 ± 2.3%, respectively) (*p* < 0.05) (Fig. 4a). Moreover, much larger numbers of cancer cells exhibiting chromosome 7 amplification (copy number >4/cell), on which the *MDR1* gene is located, were observed in the *TP53* mutant group (2.1 ± 0.7)% than in the wild-type group (0.3 ± 0.05)% (*p* < 0.01) (Fig. 4b).

### Analysis of *MDR1* copy number and expression in recurrent ovarian cancer tissues

**Fig. 5:**
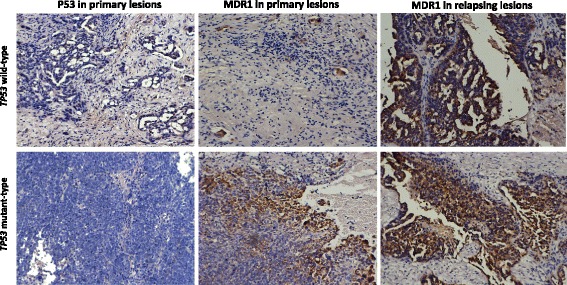
Changes in MDR1 expression in primary and recurrent lesions between the *TP53* wild-type and mutant groups detected by immunohistochemistry. First row: *TP53* wild-type group, second row: *TP53* mutant group. The first column indicates P53 expression, the second column indicates MDR1 expression in primary lesions, and the third column indicates MDR1 expression in relapse tissues. (×200)

Immunohistochemistry was performed on samples obtained from patients whose cancers had relapsed after complete remission following chemotherapy, and the ratio of patients exhibiting elevated expression of MDR1 in relapsing tissues compared with in the treatment-naïve lesions in the *TP53* mutant group was 88.5% (54/61 cases), which was significantly higher than the ratio for the *TP53* wild-type group (36.4%, 4/11 cases) (*p* < 0.01) (Fig. 5). In addition, the FISH results were consistent with the IHC results. The proportions of cancer cells containing 5-6 copies and >6 copies of *MDR1* in the *TP53* wild-type recurrent group were 36.0 ± 8.0% and 3.6 ± 0.7%, respectively, with no differences detected in the pre-treatment samples. However, in the mutant group, the proportions of cells containing 5-6 copies and >6 copies of *MDR1* were 46.0 ± 10.3% and 14.2 ± 2.7%, which were significantly higher than the proportions of cells in the corresponding primary tissues. Furthermore, in the *TP53* mutant relapse group, the proportion of cancer cells with >4 copies of chromosome 7 was significantly higher (5.4 ± 0.8)% than the proportion before recurrence (*p* < 0.01). In comparison, there was no obvious change for the copy of chromosome 7 in the wild-type relapse group (0.5 ± 0.07)%. Centromere FISH analysis revealed the accumulation of chromosome 7 in *TP53* mutant patients during cancer evolution.

## Discussion

Multi-drug resistance contributes to ovarian cancer relapse following the initial chemotherapy response [29, 30]. Despite of increased doses of first-line chemotherapy drugs (such as platinum and paclitaxel) or the application of second-line chemotherapy drugs (such as topotecan, gemcitabine, liposomal doxorubicin and docetaxel), patient prognosis is poor and the average duration of survival is less than 1 year [31, 32]. Aberrant *MDR1* expression has been observed in most chemoresistant tumors. A better understanding of the molecular mechanism linking *MDR1* and *TP53*, the most common mutated gene in ovarian cancer, may facilitate accurate prediction of the efficacy of standard chemotherapy and aid in the formulation of a rational, individualized protocol for preventive interventions in selected patients to reduce relapse and improve 5-year survival.

In the current study, we validated the use of immunohistochemical analysis to identify the presence of *TP53* mutation in ovarian cancer, in accordance with previously published data from Yemelyanova [26]. *TP53* mutation closely correlated with immunohistochemical staining patterns for P53 (strong and diffuse expression and complete lack of expression). P53 overexpression, as revealed by immunostaining, was associated with missense or frameshift mutations in *TP53*, whereas nonsense mutations resulted in protein truncation and a complete lack of immunostaining. Importantly, in addition to non-mutant *TP53* tissue, some ovarian cancer samples contained a low-abundance frameshift *TP53* mutation that resulted in weak (<10%) positive or positive (10%-50%) signals for P53 staining. Such samples were generally considered as wild-type *TP53*. There are two possible explanations for this phenomenon. First, direct sequencing may not be the most accurate gene testing method (massive parallel sequencing increases the accuracy of somatic mutation detection), and thus our detection of the low-abundance mutant tissue was a coincidence. Second, the presence of a low-abundance mutation in a tiny region of tumor tissue still results in adequate P53 protein function without impairment. In previous studies, the overall sensitivity for *TP53* mutation detection based on P53 immunostaining in high-grade serous ovarian cancer was 99%, and for epithelial ovarian cancer, the accuracy of *TP53* mutation diagnosis based on immunohistochemistry was as high as 95% [26, 27].

Based on our findings, regardless of the presence of *TP53* mutations, most relapse cases were first diagnosed as stage IIb-IIIc. The reason may be that tumor reductive surgeries can not completely remove cancer cells, and the remaining tumor cells seed recurrence. Additionally, according to our analysis, the primary reason for chemotherapeutic drug resistance and the relapse of ovarian cancer was P53 dysfunction caused by a gene mutation (class I/II mutation). The pathological grades among these *TP53* mutant relapse cases were generally higher than the grades for the wild-type relapse patients, and in terms of the histotype, mutant relapse were generally associated with non-serous cancer. Regarding chemoresistant behavior, drug resistance in the wild-type group primarily occurred during the first chemotherapy treatment after surgery (based on the proportions of partial and no remission patients), while in the mutant group, chemoresistance also emerged during the second chemotherapy treatment after relapse, with the exception of the high relapse ratio during the first chemotherapy period. The subsequent chemoresistance in the *TP53* mutant group may represent adaptive evolution as a result of the selective environmental pressure on ovarian cancer cells after the first round of chemotherapy. In addition, this result is the direct evidence of accelerating tumor chemoresistance evolution once P53 function is impaired.

A relationship between the presence of *TP53* mutation and the 5-year survival of ovarian cancer patients has been reported in many studies. In a recent analysis, the hazard ratio (HR) of P53 status on survival was only 1.47 (95% CI: 1.33–1.61), and HR differed with various conditions (such as late stage and serous cancer cases). Even in late stage cases (stages III, IV), the HR for *TP53* mutation decreased to 0.91 (95% CI: 0.59–1.39), indicating a possible protective effect, and serum P53 autoantibodies [HR: 1.09 (95% CI: 0.55-2.16)] were not associated with OS [33, 34]. This result is likely attributable to the constrained P53 function in high-grade tumors and in late-stage serous ovarian cancer, a hypothesis that need to be tested in future studies. In the current study, *TP53* mutation was not an independent factor for the 5-year survival compared with other factors (stage, grade and histotype) according to our multivariate analysis. In contrast, wild-type *TP53* was a crucial factor that correlated with disease progression by preventing chemoresistance in ovarian cancer cells. Despite the proposal that *TP53* mutations impact ovarian cancer recurrence in certain studies, in current study, the in-depth delineation of *TP53* mutation and *MDR1* copy number analysis permitted us to identify a new mechanism by which recurrence of ovarian cancer is accelerated through the evolution of chemoresistance due to *TP53* mutation. Once *TP53* is mutated, cancer cells with DNA damage are not able to activate the apoptosis program. Instead, cells remain in the G1 or G2 phase in anticipation of DNA repair [35, 36], enabling cancer cells to augment their drug resistance capacity. Notably, cancer cell DNA content and nucleus size were markedly different in the *TP53* mutant and wild-type groups, reflecting remarkable increases in the amounts of cells exhibiting polyploid and aneuploid DNA in the *TP53* mutant group, which resulted in exacerbated genomic instability and a high degree of malignancy. Furthermore, we detected larger numbers of cancer cells with >6 *MDR1* copy numbers and >4 copies of chromosome 7 in the *TP53* mutant group. Regardless of the chemotherapeutic choice, *MDR1* copy number in the mutant group was higher than the wild-type group. Based on these results, we predicted that cancer cells with high *MDR1* copy numbers that were confronted with a selective environmental pressure (for example, chemotherapy after surgery) were more likely to survive and seed recurrence. To investigate this hypothesis, we compared *MDR1* copy numbers and MDR1 expressions in original and relapse cancer tissues. The results were as expected: MDR1 protein levels were noticeably increased in relapse tissues, particularly in the *TP53* mutant patients. Upon selective pressure exerted by chemotherapy, the number of *MDR1* copies was remarkably upregulated in the *TP53* mutant group, conceivably caused by the accumulation of chromosome 7. Conversely, *MDR1* copy numbers in relapse patients in the *TP53* wild-type group were not altered, and less chemoresistance was associated with second-round chemotherapy. We attributed recurrence in the wild-type group to other factors, such as late stage or large residual lesions. Our findings confirmed that *TP53* mutation displayed an independent effect on ovarian cancer recurrence after complete remission due to effective chemotherapy. Thus, the detection of *TP53* mutation is equally as important as clinical prognostic indicators (FIGO stage, histotype, grade) for predicting ovarian cancer recurrence.

## Conclusion

The presence of *TP53* mutation in ovarian cancer exacerbates genomic instability and promotes the expression of *MDR1*, which subsequently activates chemoresistance. For these patients, particularly those with an earlier stage (stages IIb and IIc) of cancer, non-serous cancer or a lower grade (G1、G2) of cancer, the administration of high-dose chemotherapeutics during the first round of chemotherapy should be considered to prevent cancer cells from rapidly generation of chemoresistance leading to short-term recurrence.

## Abbreviations

BRCA1: Breast cancer 1
BSA: Bovine serum albumin
CEN: Centromere
FISH: Fluorescence in situ hybridization
HE: Hematoxylin and eosin
IHC: Immunohistochemistry
MDR1: Multi-drug resistance 1
OS: Overall survival
PFS: Progression-free survival
PKM2: Pyruvate kinase isoform M2
RAD17: Homolog of the Schizosaccharomyces pombe cell cycle checkpoint gene
TP: Paclitaxel and cisplatin
TP53: Tumor protein P53
